# Spatial Attention-guided Hybrid Deep Learning with Sharpened Cosine Similarity for Accurate Chest X-ray Interpretation

**DOI:** 10.2174/0115734056396626251105115902

**Published:** 2025-11-28

**Authors:** O Saraniya, B Elakkiya, Naga Sai Akhila Gurre

**Affiliations:** 1 Department of Electronics and Communication Engineering, Government College of Technology, Coimbatore, Tamil Nadu, India; 2 Department of Electronics and Communication Engineering, Sri Venkateswara College of Engineering, Chennai, Tamil Nadu, India; 3 California State University, Dominguez Hills, Carson, United States of America

**Keywords:** Variational spatial attention, Lesion segmentation, Sharpened cosine similarity, Chest X-ray, COVID-19, SARS-CoV-2

## Abstract

**Introduction:**

Life-threatening respiratory conditions such as COVID-19 and pneumonia demand rapid and accurate diagnosis. Chest X-rays (CXR) are widely used due to their accessibility and cost-effectiveness, but interpreting them remains clinically challenging, especially with overlapping radiological features.

**Methods:**

The proposed VSAG-HDL Net, a novel hybrid deep learning framework designed to enhance the accuracy and interpretability of CXR-based diagnosis. The architecture integrates a Variational Spatial Attention Fusion U-Net (VSA-FU-Net) for lesion segmentation and a Sharpened Cosine Similarity (SCS) Network for disease classification. A dataset of 21,165 CXR images from the Radiography Database was used. Segmentation performance was evaluated using Dice Similarity Coefficient (DSC) and Intersection over Union (IoU), while classification performance was assessed *via* accuracy metrics.

**Results:**

The VSA-FU-Net achieved a DSC of 90% and an IoU of 95%, indicating high precision in localizing lesions of varying shapes and sizes. The classification module reached an overall accuracy of 95.5%, outperforming traditional CNN-based methods such as CoroDet (+4.3%), CovXNet (+5.3%), and ShuffleNet (+3.9%). Although slightly less accurate than DenseNet+VIT (–2.0%) and DenseNet+VIT+GAP (–2.3%), the proposed framework offers competitive accuracy with significantly reduced model complexity.

**Discussion:**

The elimination of redundant feature extraction and the integration of spatial attention enhance both the diagnostic performance and computational efficiency, making the framework suitable for real-world clinical settings.

**Conclusion:**

VSAG-HDL Net provides a robust, interpretable, and resource-efficient solution for chest disease detection in CXR. Its clinical integration can support early and accurate diagnostic decision-making, particularly in resource-limited environments.

## 1 INTRODUCTION

COVID-19 is a contagious illness caused by the virus known as Severe Acute Respiratory Syndrome Coronavirus 2 (SARS-CoV-2) [1]. First detected in Wuhan in late 2019, COVID-19 was declared a global health crisis by the WHO in 2020 [2]. As of December 2023, the disease has resulted in over 772 million confirmed cases and nearly 7 million deaths worldwide. According to the World Health Organization (WHO), reverse transcription polymerase chain reaction (RT-PCR) remains the benchmark for diagnosing and screening COVID-19. Despite its widespread use, RT-PCR has limitations, including extended processing times that can take up to 48 hours, limited sensitivity (approximately 60–70%), and a considerable likelihood of false negatives [3]. Due to the urgent need for fast and accurate detection to curb the spread of the virus and facilitate effective treatment, medical imaging techniques such as chest X-rays (CXR) and computed tomography (CT) scans have been explored as alternative diagnostic methods. Both CT and CXR imaging can reveal visual indicators linked to COVID-19 infection. However, CT scans come with several limitations: they are non-portable, increasing the risk of virus transmission in centralized imaging areas; they expose patients to higher radiation doses; they are more expensive and require specialized operators; and their availability is limited, especially in rural and low-resource settings [4].

• In contrast, CXR imaging has gained recognition as a valuable diagnostic tool for COVID-19 and other chest-related conditions, including pneumonia and lung opacity. Its advantages include being more accessible, cost-efficient, quicker to administer, and better suited for large-scale screening. Given these benefits, CXR plays a crucial role in identifying respiratory diseases that significantly contribute to global morbidity and mortality [5]. Although CXR imaging offers numerous advantages in detecting respiratory diseases, interpreting these images remains a complex task. One of the main challenges is the superimposition of anatomical and tissue structures along the projection path, which can obscure critical findings. Accurate interpretation demands a high level of expertise, focus, and clinical experience. With the growing reliance on CXR imaging and the substantial volume of scans generated daily, radiologists face increased workloads, which can contribute to diagnostic errors. It is estimated that real-time interpretation errors occur in approximately 3–5% of cases during routine clinical practice.

• Lesion Boundary Ambiguity - Lung conditions often exhibit overlapping radiological features, making it challenging to delineate the boundaries of infected regions accurately. In clinical practice, the ambiguity in identifying lesion boundaries is a critical challenge that can lead to both under-segmentation and over-segmentation, compromising the reliability of subsequent analyses [6, 7].

• Classification Uncertainty - Diseases such as viral pneumonia, lung opacity, and COVID-19 share common visual characteristics (*e.g*., ground-glass opacities and consolidations), leading to high inter-class similarity. This complicates classification, increasing the risk of misclassification and emphasizing the need for a precise and robust model [8, 9].

• Imaging Artifacts and Variability - CXRs are susceptible to various imaging artifacts, including noise, poor contrast, and anatomical overlaps, which often compromise segmentation and classification accuracy. In clinical settings, these artifacts can vary significantly between machines and patients, further complicating the task of segmenting lesions and accurately identifying disease regions [10, 11].

These limitations underscore the crucial need for automated and intelligent systems to support radiologists in enhancing accuracy, consistency, and diagnostic efficiency. In recent years, the scientific community has focused on developing cost-effective, automated methods, particularly for COVID-19 diagnosis and assessment, that utilize CXR images to support radiologists in delivering fast and accurate evaluations. Biomedical image processing has made significant strides in recent years, with deep learning emerging as the dominant approach for medical image segmentation tasks. Within this domain, lung segmentation has attracted considerable attention, as researchers strive to address challenges such as anatomical variability, lesion heterogeneity, and the need for computational efficiency. One influential method is the U-Net architecture, introduced by Islam *et al*. [12], which effectively balances local and global feature extraction through its encoder-decoder design. Building upon advances in segmentation, machine learning and deep learning have become central to detecting chest diseases from CXR images. Brima *et al*. [13] proposed a fast and computationally efficient deep transfer learning framework for accurate early diagnosis of COVID-19 and other pneumonia types from chest X-ray images, achieving 94.0% test accuracy. Bashar *et al*. [14] developed a framework combining image enhancement, data augmentation, and transfer learning for automatic COVID-19 pneumonia diagnosis from chest X-rays, achieving 95.63% accuracy and outperforming existing methods. However, many of these algorithms have been developed and evaluated on relatively small datasets, often limiting their generalizability and clinical applicability. To address this challenge, the COVID-19 Radiography Database was released in 2021, offering a large-scale collection of 3,616 COVID-19 images, 10,192 normal cases, 6,012 lung opacity (non-COVID) cases, and 1,345 viral pneumonia cases. This extensive dataset has enabled researchers to train and evaluate models on a broader range of classes, improving diagnostic capabilities and model reliability [15].

In 2020, Chen *et al*. [16] proposed a Two-Stream Collaborative Network (TSCN) for multi-label chest X-ray classification, combining global image and lung field features using U-Net lung segmentation. The method improved overall AUC to 0.816, outperforming other models in detecting nodules, pneumothorax, and emphysema. However, its performance on infiltration was limited due to the complexity of the textures. In 2021, Vidal *et al*. [17] proposed a two-stage transfer learning approach that adapted a pre-trained brain MRI segmentation model to identify lung regions in portable chest X-rays, achieving high accuracy in classifying COVID-19 (0.9761), normal (0.9801), and related pulmonary conditions (0.9769), even with limited and low-quality data. In 2022, Maity *et al*. [18] proposed a DCNN-based method with residual links and advanced preprocessing (Top-Hat/Bottom-Hat, CLAHE) to improve lung segmentation in chest X-rays for CAD systems. Tested on different datasets, it achieved a Dice score of 0.982 and a Jaccard score of 0.967, outperforming existing methods while maintaining a lightweight architecture suitable for standard GPUs. In 2018, Dai *et al*. [19] proposed SCAN, a dual-network framework comprising a Fully Convolutional Network (FCN) and a critic network. SCAN refines segmentation masks through adversarial training, ensuring alignment with the ground truth.

In 2022, Ukwuoma *et al*. [20] developed the Inceptionv3 model by incorporating light-chroma separation using global second-order pooling instead of average pooling, achieving 98.2% accuracy with efficient computation. Khan *et al*. [21] introduced a multi-class classification method that combined multiple pre-trained models with a custom classification head. Using data augmentation to balance the dataset, their EfficientNetB1 model reached a top accuracy of 96.13%. Meanwhile, Pan *et al*. [22] proposed a deep neural network (DNN) with multiple channels that utilizes feature fusion, achieving an average classification accuracy of 93.19% across four categories. Liu *et al*. [23] improved the U-Net model by incorporating residual links and a tailored leaky activation function to enhance lung segmentation in chest X-rays. It performed well on poor-quality images and abnormal cases, such as pleural effusion; however, the added residual blocks increased processing time, affecting real-time application. Ahila *et al*. [24] proposed the E-GCS network, a multi-modal framework designed to differentiate normal and abnormal COVID-19 cases using ultrasound, X-ray, and CT scan images. The model integrates an Attention Bottleneck Residual Network (AB-ResNet) to prioritise diagnostically relevant features across modalities, improving cross-domain generalization.

Pal *et al*. [25] introduced a segmentation architecture for detecting small lesions in medical images. The model combines dense convolutional layers in an encoder-decoder structure (UW-Net) with skip connections enhanced by attention mechanisms. These skip connections refine feature propagation, enabling precise localization of subtle lesions often missed by conventional U-Net variants. Tang *et al*. [26] proposed an X-ray lung segmentation framework that enhances data augmentation with synthetic pathological images and improves boundary delineation using a Crisscross Attention module. Singh *et al*. [27] developed a computationally efficient lung segmentation model leveraging the DeepLabv3+ architecture. The model strikes a balance between accuracy and efficiency, leveraging MobileNetV2’s parameter-efficient design for real-time segmentation while maintaining competitive performance on benchmark datasets.

Gupta *et al*. [28] proposed a hybrid framework that combines Attention U-Net for lung segmentation and Vision Transformers (ViTs) for the classification of chest X-rays. The study aimed to enhance performance and explainability in the diagnosis of lung diseases. The model achieved a DSC of 98.54%, while MobileViT reached 98.52% accuracy, outperforming CNNs by 9.26%. The use of Grad-CAM++ and Layer-wise Relevance Propagation provided transparency into the model's decision-making process, promoting clinical trust. Geng *et al*. [29] introduced STCNet, a hybrid segmentation network that integrates Swin Transformers and CNNs to accurately segment regions of scattered lung infections in COVID-19 CT images. The model incorporates a ReSwin transformer block for richer feature extraction and a skip connection cross-attention module to preserve boundary information. Additionally, a scale-aware pyramid fusion module enhances the integration of multi-scale context. STCNet achieved Dice scores of 79.92% and 82.78% on two benchmark COVID-19 datasets, outperforming existing methods in infection segmentation. Ağralı *et al*. [30] proposed U-TranSvision, a transformer-based model for segmenting lesions in chest CT images. To enhance the detection of small lesions, the model integrates salient feature learning and utilizes Pix2Pix GAN for data augmentation. Noise removal preprocessing was applied, and a new dataset of 11,717 annotated CT slices was introduced. U-TranSvision achieved a Dice Similarity Coefficient of 85.57% and an IoU of 74.82%, demonstrating superior performance and robustness on multiple public datasets (COVID-19-CT-Seg, MosMedData, and MedSeg) while maintaining low computational complexity.

Despite notable advances in deep learning (DL) for chest X-ray (CXR) analysis, many existing studies are constrained by simplified classification tasks, often involving binary or three-class problems focusing on COVID-19, pneumonia, and normal cases. These models are frequently trained on relatively small or artificially augmented datasets, which can limit generalizability and introduce biases that are not representative of real-world clinical data. Furthermore, inconsistencies in data partitioning and limited transparency in experimental protocols have raised concerns about reproducibility and robustness in reported findings [31].

To overcome these challenges, we propose VSAG-HDL Net, a Variational Spatial Attention-Guided Hybrid Deep Learning framework that combines segmentation and classification in a unified architecture. This approach addresses the need for both anatomical precision and contextual understanding by integrating spatial attention mechanisms and sharpened cosine similarity for improved feature discrimination [32-34]. By coupling segmentation with classification, VSAG-HDL Net leverages structural cues from lung regions to enhance class-specific pattern recognition. This design supports more accurate differentiation between COVID-19, viral pneumonia, lung opacity, and healthy cases on large, publicly available CXR datasets, providing a comprehensive and interpretable solution for real-world clinical applications.

Specifically, VSAG-HDL Net enhances CXR analysis through:

• **VSAFU-Net**, which integrates spatial and channel attention mechanisms to achieve enhanced segmentation accuracy. This design enables precise refinement of lesion boundaries and improves the localization of infected lung regions on chest X-rays.

• **SCS**, which improves classification performance by enhancing feature discrimination and minimizing inter-class similarities. This contributes to more reliable differentiation between similar diseases.

• The proposed approach outperforms traditional methods in lesion segmentation and disease classification on diverse CXR data, while providing interpretable and reliable outputs through attention-based visualization and explainability techniques, enhancing clinical workflows with accurate and transparent multi-disease diagnosis.

## 2 MATERIALS AND METHODOLOGY

### 2.1 Radiography Dataset

**Fig. (1):**
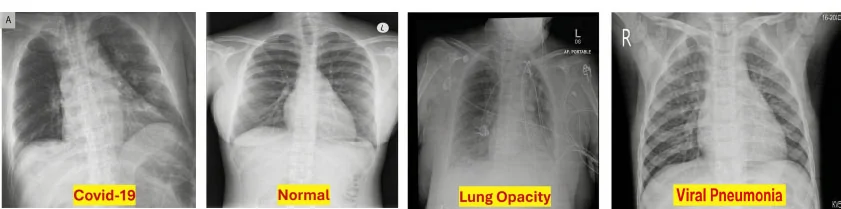
Sample chest X-ray images for different disease categories.

The Radiography Dataset is a publicly available collection of 21,165 chest X-ray images, curated to support research on lung disease classification and segmentation [35]. As outlined in Table **1**, the dataset is divided into four classes: COVID-19 (3,616 images, 17.08%), Viral Pneumonia (1,345 images, 6.36%), Lung Opacity (6,012 images, 28.40%), and Normal (10,192 images, 48.16%). Each image is accompanied by a corresponding segmentation mask, enabling both classification and segmentation tasks. Figure **1** shows sample images from the dataset.

As detailed in Table **2**, the dataset is divided into training, validation, and testing subsets for balanced model training and evaluation.

**Fig. (2):**
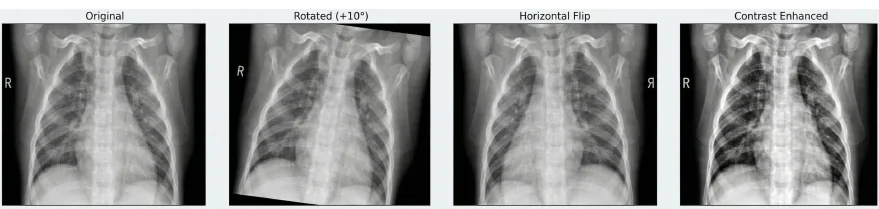
Augmented chest X-ray images with different transformations.

Before training the deep learning models, all images undergo standardized preprocessing steps to ensure data consistency and improve model performance. The following preprocessing techniques are applied:

• **Image Resizing:** All chest X-ray (CXR) images are resized to 256 × 256 pixels to standardize input dimensions across the model. This resizing ensures compatibility with common deep learning architectures while preserving essential anatomical features, particularly in the lung regions.

• **Normalization:** Pixel values are scaled to a range of 0–1 using min-max normalization. This step improves model stability during training by preventing numerical issues and facilitates faster and more consistent convergence [36].

• **Data Augmentation:** Data augmentation strategies were used to reduce class imbalance and strengthen the model's ability to generalize. Techniques such as rotation (±10°), horizontal flipping, and contrast enhancement [37]. These specific augmentations are chosen because they simulate natural variations in clinical imaging, such as different patient positions, orientations, and imaging conditions, without altering the fundamental diagnostic features. Introducing such variability makes the model more robust to real-world data inconsistencies and less likely to overfit the training set. Figure **2** shows the sample augmented images.

### 2.2 Methodology

#### 2.2.1 Variation Spatial Attention Fusion U-Net for Lesion Segmentation

The Variational Spatial Attention Fusion U-Net (VSAFU-Net) is a novel encoder-decoder architecture tailored to segmenting complex thoracic pathologies from chest radiographs. Built upon the foundational U-Net framework, VSAFU-Net enhances segmentation performance by integrating a Variational Spatial Attention Gate (VSAG), which enables dynamic feature refinement by modeling spatial uncertainty in lesion representation. Figure (**3**) illustrates the complete architecture of the proposed network.

##### 2.2.1.1 Architecture Overview

VSAFU-Net consists of four primary components: a pretrained Inception feature extractor, a hierarchical encoder, variational spatial attention gates bridging the encoder and decoder pathways, and a multi-stage decoder for segmentation map reconstruction.

➢ The encoder path comprises four convolutional stages, each consisting of two convolutional layers followed by a ReLU activation and max pooling. The final encoder block includes a dropout layer to mitigate overfitting due to high feature complexity at deeper levels.

➢ Inception modules, pretrained on large-scale datasets, are embedded before the encoder to extract high-level semantic features. This facilitates the network’s capacity to handle diverse and subtle manifestations of pulmonary abnormalities, such as COVID-19-related opacities, viral pneumonia, and diffuse lung pathologies.

➢ The decoder path mirrors the encoder in structure and employs upsampling operations to reconstruct the spatial resolution of the segmentation map progressively. Each decoder block receives concatenated input from its corresponding encoder stage *via* a VSAG module.

##### 2.2.1.2 Variational Spatial Attention Gate (VSAG)

A core innovation of VSAFU-Net is the Variational Spatial Attention Gate (VSAG), positioned between each encoder and decoder block. Unlike traditional skip connections that pass features indiscriminately, the VSAG mechanism filters encoder outputs by estimating spatial attention maps through variational inference. Each Variational Spatial Attention Gate (VSAG) models the spatial attention map *A*(*x*) as a stochastic process, formulated in Eq. (**1**), where attention is sampled from a learned distribution to reflect spatial uncertainty:

**Table:** 

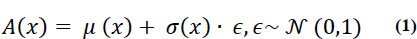

Where *µ*(*x*) and *σ*(*x*) are the mean and standard deviation maps, predicted by parallel convolutional branches.

The resulting attention maps *A*(*x*), derived *via* variational inference, are then employed to modulate the encoder feature maps *F*(*x*) through element-wise multiplication, as described in Eq (**2**):

**Table:** 

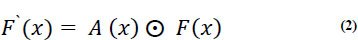

In this equation, *F’*(*x*) represents the refined feature maps, and 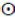 denotes the Hadamard (element-wise) product. This modulation mechanism enhances the network’s focus on spatially relevant regions, allowing it to prioritize diagnostically significant features while suppressing background noise.

During training, VSAFU-Net minimizes a hybrid loss function designed to simultaneously improve segmentation accuracy and promote meaningful spatial attention through uncertainty regularization. The total loss 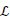 is composed of two terms: the primary segmentation loss 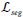 and the Kullback-Leibler (KL) divergence term 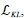, which regularizes the variational spatial attention mechanism. This is expressed using Eq (**3**):

**Table:** 

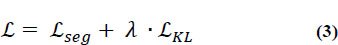

Here, *λ* is a weighting hyperparameter that balances the contribution of the KL divergence relative to the segmentation loss.

The KL divergence term penalizes deviation from the standard normal distribution, encouraging the learned attention weights to remain interpretable and well-behaved. It is defined using Eq. (**4**):

**Table:** 

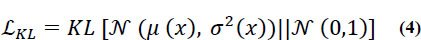

This formulation ensures that the attention distributions are regularized while enhancing segmentation fidelity. In addition to spatial attention gates, the architecture incorporates a scale-aware fusion module in the decoder to aggregate multi-scale features. This module balances low-level texture details with high-level semantic content, enabling the model to handle both fine-grained edges and coarse infection patterns. Such fusion is particularly beneficial in chest X-rays, where infection regions can be diffuse and overlapping. This architecture improves robustness to overlapping pathologies and low-contrast regions, achieving superior performance in tasks such as COVID-19 lesion segmentation. By integrating probabilistic attention and uncertainty quantification, VSAFU-Net enhances segmentation precision and provides interpretable outputs to support clinical decision-making.

#### 2.2.2 Theoretical and Empirical Advantages of SCS-Net over Conventional CNNs

Traditional CNNs rely on learned filters applied *via* dot product operations to extract spatial hierarchies of features. While effective, these filters are sensitive to scale, orientation, and translation, often requiring extensive data augmentation and complex network depth to generalize well. Moreover, the dot product can yield strong activations for patterns with large magnitudes, even when their orientations do not align with the learned filters, potentially weakening feature selectivity. To address these limitations, the proposed SCS-Net replaces convolutional layers with SCS layers, which compute feature similarity using a normalized dot product between input patches and template kernels. The SCS operation evaluates the cosine of the angle between two vectors, image patch X and kernel Y, as, shown in Eq (**5**):

**Table:** 

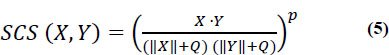

Here, Q is a small constant for numerical stability, and P is a learnable exponent that sharpens the response by amplifying differences in angular alignment.

**Fig. (4):**
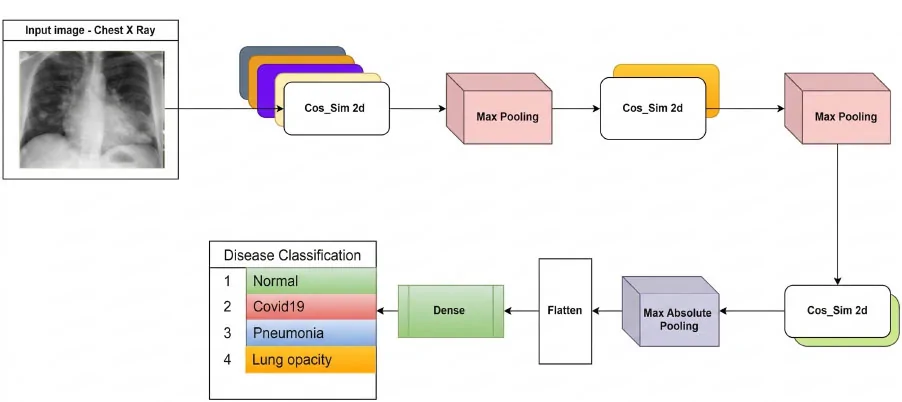
Sharpened Cosine Similarity (SCS) architecture.

Unlike standard convolutions, this method discards the absolute magnitude. It emphasizes directional similarity, thereby offering robust discrimination even in cases of subtle inter-class variations, such as those seen in CXR images, which differentiate COVID-19 from other pneumonias. The sharpening exponent *P* serves to heighten sensitivity to alignment discrepancies. Minor angular deviations corresponding to visually similar but pathologically distinct patterns are significantly magnified, improving the network’s ability to isolate key disease characteristics. Furthermore, replacing traditional max-pooling with absolute max-pooling allows the network to retain the most intense activations, regardless of sign, preserving clinically meaningful signals that may otherwise be lost. Figure **4** depicts the SCS-Net architecture with layers combined. From a computational standpoint, SCS-Net is parameter-efficient, reducing the number of trainable weights by 10x to 100x compared to standard CNNs, as shown in Fig. (**5**). This simplifies training, minimizes the risk of overfitting on small datasets, and enhances the ability to deploy in real-time in resource-constrained clinical settings. Empirical results confirm that SCS-Net outperforms conventional CNN-based models across several metrics (accuracy, precision, recall, F1-score, and Sensitivity) in multiclass classification tasks.

#### 2.2.3 VSAG-HDL Net Model Workflow

The proposed VSAG-HDL Net framework, shown in Fig. (**6**), introduces a comprehensive and systematic three-stage deep learning pipeline tailored for precise and reliable chest X-ray image analysis. In Stage 1, rigorous preprocessing is performed to ensure consistency and robustness across the input data. Raw chest X-ray radiographs are first resized to a standardized resolution, allowing the model to process images with uniform spatial dimensions. Intensity normalization is subsequently applied to rescale pixel values into a consistent range, mitigating the effects of variability introduced by differing X-ray machines or acquisition settings. To further enhance the model’s generalizability and address the prevalent issue of class imbalance, extensive data augmentation techniques, including random rotations, horizontal and vertical flipping, and contrast modulation, are explicitly applied to the training set. This augmentation strategy not only enriches the diversity of training samples but also ensures that the model is exposed to a wide range of anatomical and pathological variations. The dataset is then partitioned into training (80%), Validation (10%), and testing (10%) subsets to facilitate practical model training and evaluation.

In Stage 2, the preprocessed images are input into the Variational Spatial Attention Fusion U-Net (VSAFU-Net), which performs precise lesion segmentation. This module is built on an encoder-decoder U-Net architecture enhanced with variational spatial attention (VSA) mechanisms embedded in the skip connections. The encoder extracts hierarchical, multi-scale features through successive convolutional and max-pooling layers, capturing both local textures and global structural information. The decoder, on the other hand, uses transposed convolutional layers to upsample and reconstruct high-resolution segmentation maps. The VSA modules play a critical role by learning to probabilistically emphasize spatial regions with high diagnostic relevance while suppressing irrelevant or noisy background features. These modules incorporate variational inference to model spatial uncertainty and dynamically adjust attention weights during training, resulting in more reliable and interpretable segmentation. The model is trained using a hybrid loss function that combines Dice loss, which ensures the accurate delineation of segmented areas, and KL divergence, which promotes effective regularization and minimization of uncertainty.

Stage 3 focuses on disease classification using the SCS Net, which diverges from CNNs by utilizing a unique similarity-based approach. In SCS Net, conventional convolutional layers are replaced by SCS layers, which compute normalized dot products between feature vectors and then apply a sharpening exponent to enhance the discriminative capacity. This design significantly reduces the number of trainable parameters while improving sensitivity to subtle inter-class variations, which is particularly beneficial when distinguishing visually similar conditions, such as COVID-19, pneumonia, and lung opacities. During training, the SCS Net is fine-tuned using a combination of cross-entropy loss and SCS-specific regularization, which collectively mitigate overfitting and encourage robust feature learning. The model operates directly on the segmentation masks generated in Stage 2, eliminating the need for manual feature engineering. Evaluation is conducted through standard performance metrics, ensuring a comprehensive assessment of the model’s predictive capabilities. Furthermore, iterative model refinement is employed based on evaluation results, allowing for adaptive tuning of hyperparameters and architectural components to enhance performance across diverse clinical environments.

#### 2.2.4 Evaluation Metrics

The model’s performance was assessed using standard evaluation metrics derived from the confusion matrix, which used the dataset’s class labels as a reference. An example demonstrating the model’s classification results for the lung opacity class is shown in Fig. (**7**). The corresponding evaluation metrics, including accuracy, precision, recall, specificity, and F1-score, were computed using the formulas provided in Eqs. (**6**-**10**).

**Table:** 

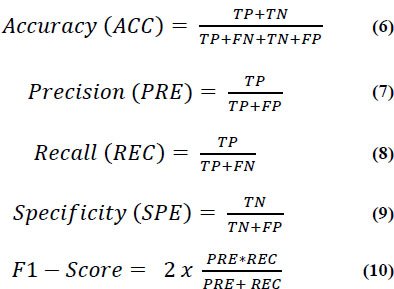

## 3 EXPERIMENTAL SETUP AND EVALUATION

The proposed VSAG-HDL Net model was implemented and trained using the Kaggle GPU environment, which provides access to NVIDIA Tesla P100 GPUs. This platform was chosen for its availability of free computational resources, ease of use, and reproducibility. Although we did not benchmark it against other platforms, it enabled efficient training and experimentation within our resource constraints. Grid search-based hyperparameter tuning was employed to optimize model performance by systematically exploring combinations of learning rate, batch size, optimizer type, and loss functions. The batch size was tested at 16, 32, and 64. A batch size of 32 was selected as it provided a good trade-off between stable gradient updates and GPU memory efficiency. A batch size of 64 caused memory overflow issues, while 16 resulted in slower convergence without notable performance gains. Learning rates of 0.1, 0.01, 0.001, and 0.0005 were evaluated. A value of 0.001 was found to be optimal, offering a balance between fast convergence and stable training. Higher learning rates caused oscillations in training loss, whereas lower rates significantly delayed convergence. Among the evaluated optimizers, SGD, RMSprop, and Adam, Adam proved to be the most effective, yielding faster convergence and lower validation loss due to its adaptive learning rate mechanism.

Dice loss, Binary Cross-Entropy (BCE), and a hybrid were examined for the segmentation task. The combined Dice + BCE loss function demonstrated superior performance by enhancing boundary accuracy and handling class imbalance more effectively. Categorical Cross-Entropy was selected for the classification task over Focal Loss, as data augmentation sufficiently addressed class imbalance. The learning rate was dynamically adjusted using the ReduceLROnPlateau scheduler, which demonstrated superior performance compared to StepLR and fixed-rate schedules. By monitoring validation loss, it effectively adjusted the learning rate to match the training dynamics and mitigated the risk of premature convergence. All hyperparameter combinations were evaluated using an 80% training and 10% validation split of the dataset. The final model performance was assessed on the remaining 10% test set, which was strictly held out and not utilized during either the training or validation phases. The model was trained for 50 epochs with early stopping to ensure convergence without overfitting. This comprehensive grid search strategy ensured the final configuration delivered high performance in segmentation and classification tasks while maintaining computational efficiency and training stability.

## 4 RESULTS AND DISCUSSION

### 4.1 Segmentation Performance of Variational Spatial Attention Fusion U-Net

The proposed Variational Spatial Attention Fusion U-Net (VSAFU-Net) significantly outperforms existing segmentation models in accurately delineating lesion regions in chest X-ray images, as shown in Table **3**. Quantitative results demonstrate that VSAFU-Net achieves a DSC of 90.5%, an IoU of 95.0%, a sensitivity of 97.0%, and the lowest HD of 4.10 among all compared models. These results reflect the model’s superior capability in capturing both global and local contextual information, as well as precisely outlining complex lesion boundaries. In comparison, conventional architectures such as U-Net and FCN8s exhibit lower performance, with Dice scores of 78.7% and 74.1%, respectively, and higher HD values, indicating less accurate boundary predictions. The integration of variational spatial attention gates within the encoder-decoder structure allows VSAFU-Net to effectively model spatial uncertainty, enabling it to focus on diagnostically relevant regions and suppress irrelevant background features. Additionally, the hybrid loss function, which incorporates both segmentation loss and KL divergence, contributes to robust and stable training. These enhancements collectively enable VSAFU-Net to provide more accurate, interpretable, and clinically reliable segmentation results, making it a promising tool for automated multi-disease diagnosis in chest radiography.

The performance of the VSA mechanism demonstrated varying effectiveness across different chest diseases due to the unique characteristics of lesions associated with each condition. In diseases such as viral pneumonia, where lesions are well-defined and consolidated, the VSA mechanism effectively highlights regions of interest, leading to improved segmentation and classification accuracy. However, in the case of COVID-19, which often presents with subtle, heterogeneous ground-glass opacities, the attention mechanism faced challenges in distinguishing these low-contrast areas from normal lung tissue, requiring more refined training to capture these nuances. Additionally, lung turbidity (opacity) conditions, characterized by diffuse and interstitial changes, posed difficulties for the VSA mechanism due to the lack of distinct lesion boundaries. These findings suggest that while the VSA mechanism is highly effective for diseases with clear lesion boundaries, it may require further adaptation or fine-tuning for diseases with subtle or diffuse lesion characteristics. Future work will focus on refining the VSA mechanism for these challenging conditions to improve its robustness and generalization across diverse chest disease types.

These weights are optimized through variational inference, enabling the model to handle ambiguous or overlapping features with high precision. Combining Dice loss and KL divergence, the hybrid loss function ensures accurate segmentation while minimizing uncertainty in attention maps. This approach significantly reduces the HD to 4.10, indicating precise boundary delineation of infected regions shown in Figs. (**8** and **9**). The model’s ability to adaptively refine attention weights during training enhances its robustness to lesion shape, size, and location variations, making it particularly effective for complex medical tasks.

### 4.2 Classification Performance of SCS-Net

The classification performance of SCS-Net was rigorously evaluated using a multi-class dataset comprising four categories: COVID, Lung Opacity, Normal, and Viral Pneumonia. The model’s training dynamics, diagnostic accuracy, and per-class evaluation metrics are analyzed below. During training, SCS-Net demonstrated robust convergence behavior. The left panel of Fig. (**10**) shows a steady decrease in both training and validation loss, stabilizing after approximately 14 epochs, indicating effective optimization without overfitting. The right panel highlights a corresponding rise in training and validation accuracy, reaching plateaus near 97.5% and 95.5%, respectively. This alignment between training and validation metrics confirms the model’s generalization capability.

**Fig. (11):**
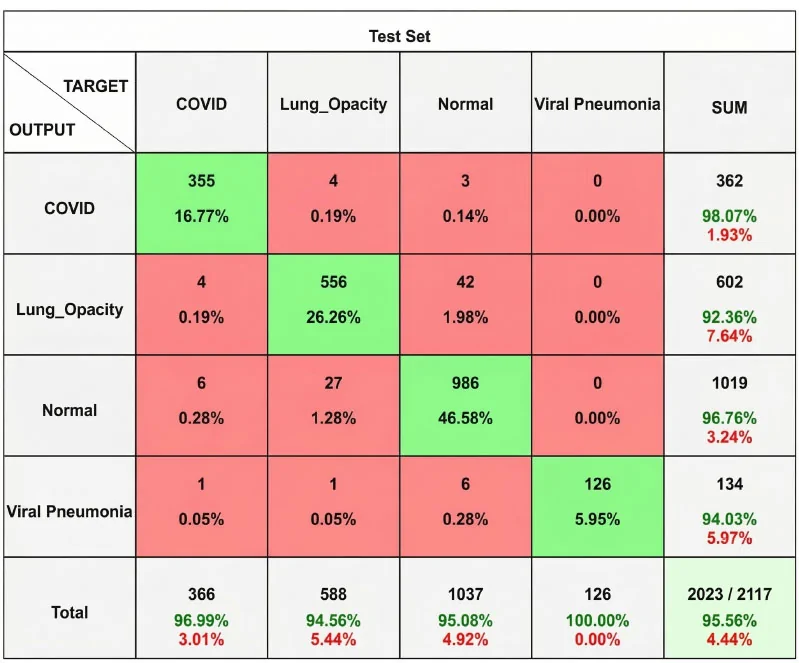
SCS net confusion matrix for test data.

Figure **11** presents the confusion matrix of the proposed model, offering detailed insights into its performance across individual classes. SCS-Net achieved high accuracy across all categories, with 98.07% accuracy for COVID, 92.36% for Lung Opacity, 96.76% for Normal, and 94.03% for Viral Pneumonia. Notably, the model exhibited minimal false positives for Viral Pneumonia (0 misclassifications) and Normal cases (0 misclassifications). However, minor inter-class confusion occurred, particularly between COVID and Lung Opacity (4 misclassified instances) and Normal and Lung Opacity (42 misclassified cases). These overlaps reflect challenges in distinguishing subtle radiographic patterns, a common issue in medical imaging tasks.

The quantitative evaluation shown in Fig. (**12**) demonstrates SCS-Net's exceptional diagnostic reliability, with consistently high-performance metrics across all classes. For COVID cases, the model achieved near-perfect precision (98.07%) and recall (96.99%), yielding an outstanding F1-score of 97.53%. Similar robustness was observed for Lung Opacity (F1=93.45%), Normal cases (F1=95.91%), and Viral Pneumonia, where the model demonstrated perfect recall (100%) combined with high precision (94.03%) for an excellent F1-score of 96.92%. With an overall accuracy of 95.56% and a remarkably low misclassification rate of just 4.44%, SCS-Net proves highly effective in clinical decision-making. The strong macro-F1 (0.9595) and weighted-F1 (0.9557) scores further highlight the model's ability to maintain balanced performance across imbalanced class distributions, making it particularly valuable for real-world medical applications where diverse pathologies must be accurately identified. These results confirm SCS-Net as a reliable, well-rounded diagnostic tool capable of handling complex clinical scenarios with high precision and sensitivity.

The experimental results demonstrate that SCS Net (VSAG-HDL Net) achieves performance metrics comparable to state-of-the-art models while maintaining architectural simplicity, as shown in Table **4**. With an accuracy of 95.5%, our approach significantly outperforms conventional deep neural networks (87.1%) and ensemble machine learning methods (87.7%), showing improvements of 4.0–8.4% across lightweight CNN architectures such as SqueezeNet (89.4%) and ShuffleNet (91.6%). Although hybrid systems combining CNNs and transformers (*e.g*., DenseNet + ViT + GAP) achieve a marginally higher accuracy (97.8%), this 2.3% gain comes at the expense of substantially greater computational demands. SCS Net’s clinical utility is further evidenced by its balanced diagnostic capabilities: it achieves 92.4% precision and 94.9% recall, surpassing specialized models like Corodet (precision: -0.4%, recall: -3.0%) and CovXNet (precision: -1.6%, recall: -5.0%). The model’s F1-score of 93.6% places it among the top performers, narrowly trailing only the aforementioned CNN-transformer hybrids (*e.g*., DenseNet + ViT+GAP: 96.2%). Notably, SCS Net demonstrates exceptional specificity (98.3%), matching the false-positive reduction capabilities of dedicated medical imaging systems, such as COVIDetection-Net (98.1%) and DenseNet + ViT+GAP (98.1%). These findings suggest that SCS Net represents a compelling compromise between diagnostic accuracy and operational efficiency. While advanced hybrid architectures achieve marginally better metrics (≤2.3% in accuracy, ≤2.6% in F1-score), their complex multi-model frameworks introduce significant implementation challenges. In contrast, SCS Net’s streamlined architecture makes it particularly well-suited for deployment in resource-constrained clinical environments where computational efficiency and rapid inference are critical.

**Fig. (13):**
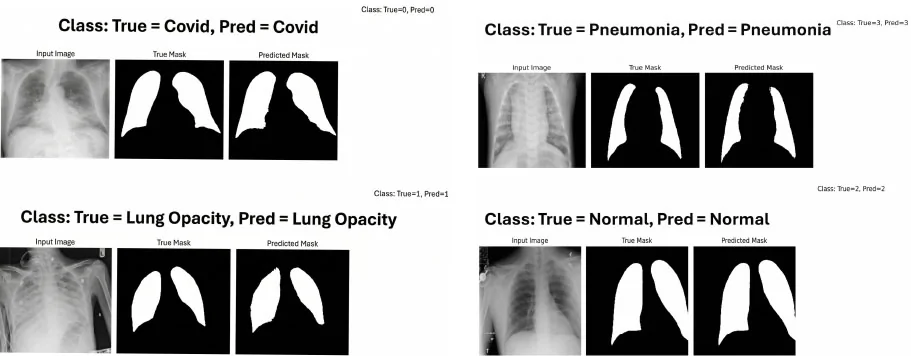
SCS Net performance on test data.

Figure **13** presents the visual classification outcomes generated by the proposed SCS-Net on representative samples from the test data. Each sample comprises the original CXR, the ground truth mask, the predicted mask, and both the actual and predicted disease labels. The results indicate that SCS-Net not only performs accurate multi-class classification but also produces segmentation masks that align closely with the ground truth across diverse pathological cases. The model correctly identified each condition with high confidence, including subtle differences in lesion patterns. Notably, in complex cases such as viral pneumonia and lung opacity, where visual features often overlap, SCS-Net effectively leveraged its sharpened cosine similarity mechanism to enhance feature discrimination and reduce inter-class ambiguity. These findings underscore the ability of the integrated hybrid framework comprising VSAFU-Net for spatially attentive segmentation and SCS-Net for robust classification to deliver interpretable and clinically meaningful predictions. The consistency between the predicted and actual disease classes highlights the framework's potential for aiding real-time diagnostic workflows.

## CONCLUSION AND FUTURE SCOPE

This study introduced VSAG-HDL Net, a hybrid deep learning framework designed to improve lesion segmentation and multi-class classification in chest X-ray (CXR) analysis. By integrating the strengths of VSAFU-Net and SCS-Net, the model addresses key diagnostic challenges, including fuzzy lesion boundaries, inter-class similarity, and imaging noise. In the segmentation stage, VSAFU-Net leverages a probabilistic spatial attention mechanism to effectively capture both global context and fine-grained features, outperforming established models such as U-Net and FCN8s. It achieved a Dice Similarity Coefficient (DSC) of 90.5%, IoU of 95.0%, and sensitivity of 97.0%, highlighting its ability to accurately localize infected lung regions, even in challenging cases with subtle manifestations. In terms of classification, SCS-Net applies sharpened cosine similarity for angular feature alignment, enhancing class separation and reducing misclassification between visually similar categories. The model achieved an overall accuracy of 95.5%, with precision of 92.4%, recall of 94.9%, F1-score of 93.6%, and specificity of 98.3%, validating its robustness and efficiency. Unlike CNN-transformer hybrids, VSAG-HDL Net maintains a lightweight architecture, enabling faster inference and lower computational cost without compromising performance. Overall, VSAG-HDL Net represents a clinically viable and computationally efficient solution for CXR-based screening. Its accurate, interpretable, and portable design supports real-time application in both high-resource hospitals and low-resource environments. Future work may explore extending the model to multimodal inputs such as CT scans and clinical metadata, refining attention mechanisms for better detection of early-stage or subtle findings, and optimizing the architecture through techniques like model pruning, quantization, and knowledge distillation to enable effective deployment on edge or mobile devices.

While the proposed model demonstrates promising performance, certain limitations remain. Misclassification can occur in cases with diffuse or early-stage abnormalities due to limited visual distinction. Additionally, the model’s adaptability across different clinical environments and imaging protocols needs further validation. Future work will focus on improving the attention mechanism *via* adaptive kernels, expanding datasets with underrepresented cases, incorporating multimodal inputs such as clinical metadata and CT scans, and applying compression techniques like pruning and knowledge distillation. These efforts will facilitate the deployment of VSAG-HDL Net in diverse real-world settings, particularly in low-resource clinical environments where speed, accuracy, and efficiency are critical.

## Figures and Tables

**Fig. (3):**
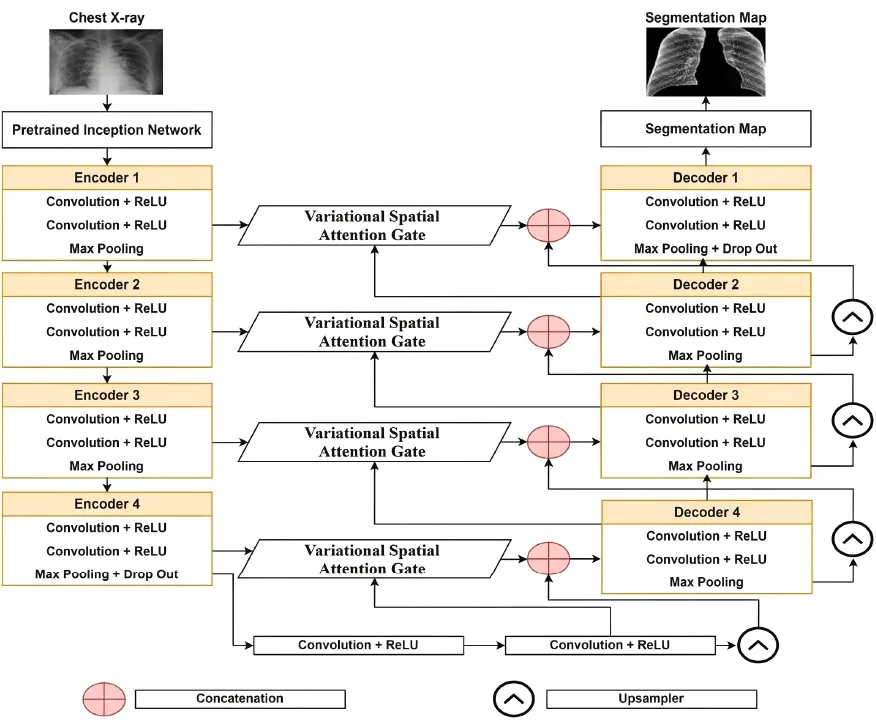
Variational spatial attention gate fusion U-Net architecture for segmentation.

**Fig. (5):**
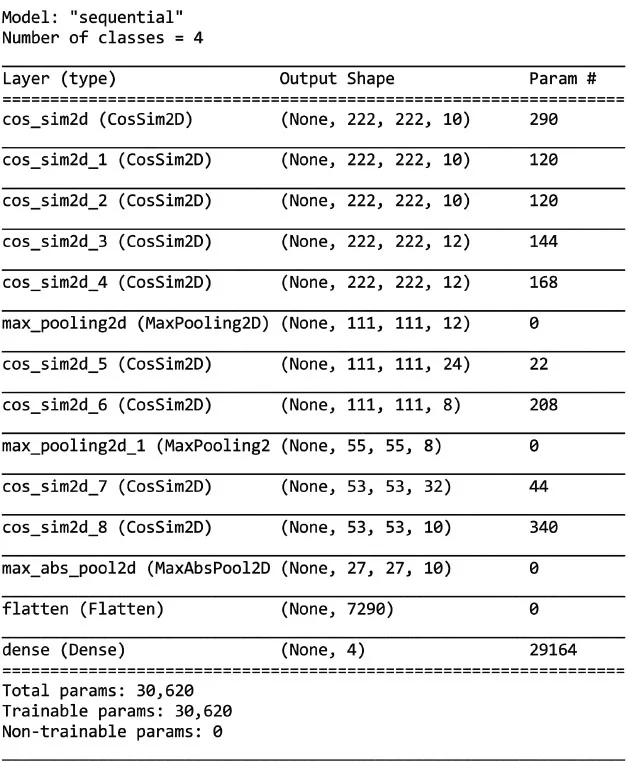
SCS-Net architecture parameters.

**Fig. (6):**
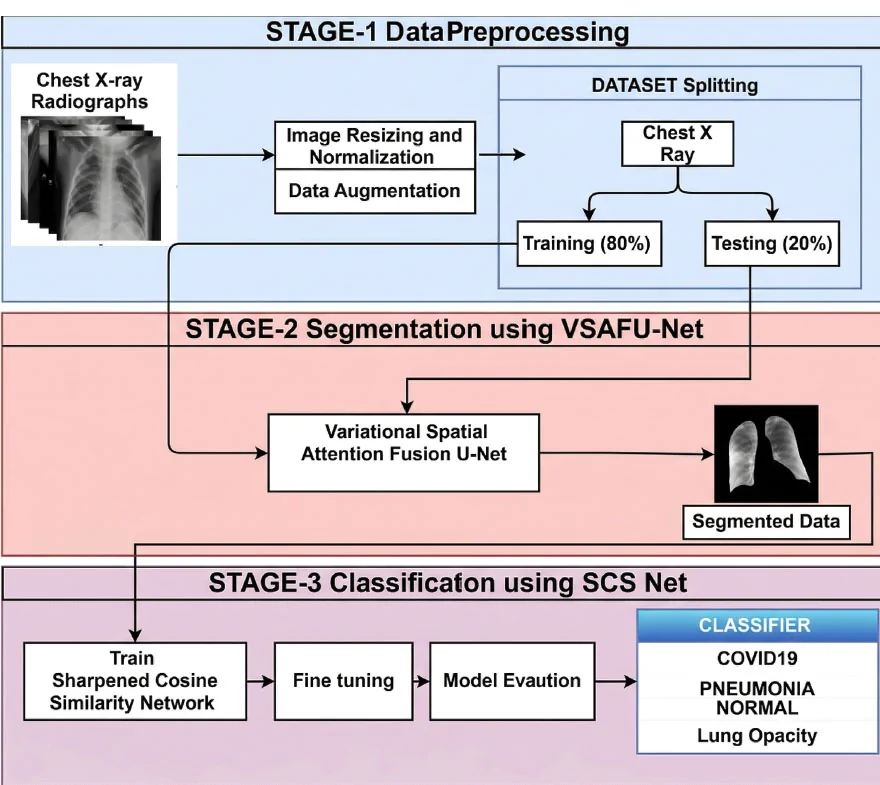
VSAG-HDL Net: A hybrid deep learning framework for chest X-ray analysis.

**Fig. (7):**
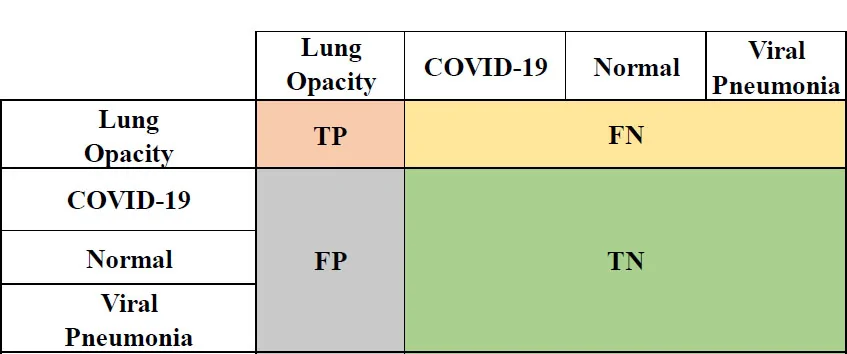
Confusion matrix for lung opacity performance evaluation.

**Fig. (8):**
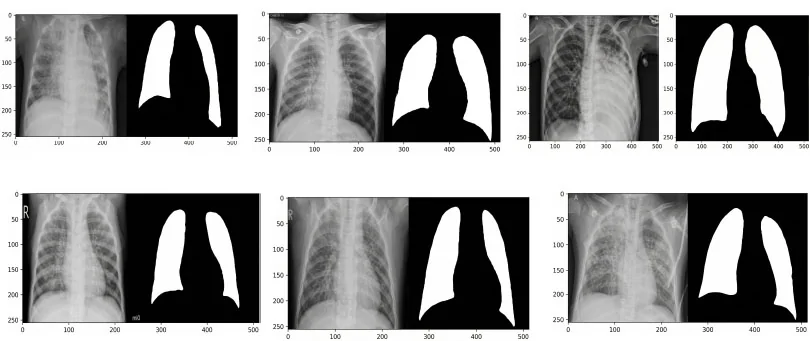
Viral pneumonia segmented image using VSAFU-Net.

**Fig. (9):**
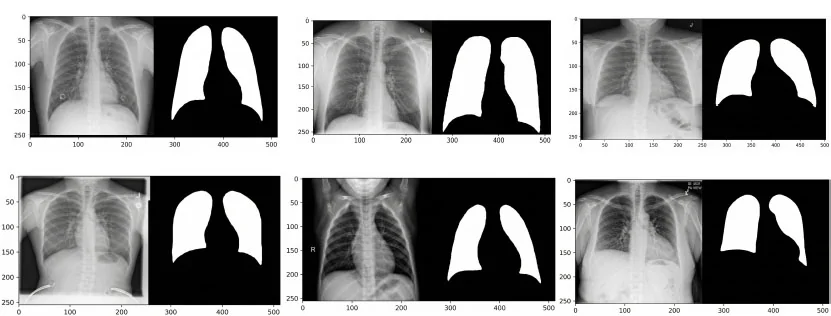
Normal lungs segmented image using VSAFU-Net.

**Fig. (10):**
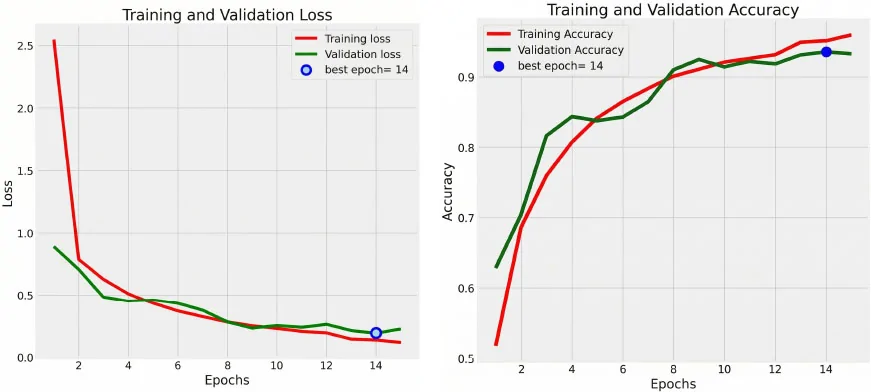
SCS model training and validation performance analysis.

**Fig. (12):**
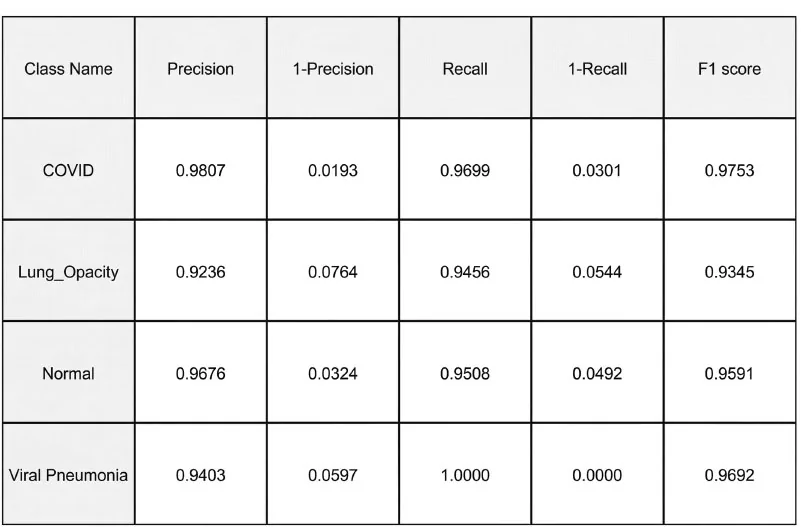
Classification performance metrics by class (Precision, Recall, F1, Accuracy).

**Table 1:** Dataset composition.

**Disease Type**	**Total Images**
COVID-19	3,616
Viral pneumonia	1,345
Lung opacity	6,012
Normal	10,192
**Total**	**21,165**

**Table 2:** Dataset split for training, validation, and testing.

**Subset**	**Sample images**	**Percentage (%)**
Training	16,932	80%
Testing	2,116	10%
Validation	2,117	10%
**Total**	**21,165**	**100%**

**Table 3:** Segmentation performance of VSAFU-Net.

**Model**	**DSC (%)**	**IoU (%)**	**Sensitivity (%)**	**HD**
CENet	83.5	72.9	93.6	4.77
SACU-Net	84.9	74.6	85.7	4.31
UNet++	80.1	68.0	94.3	5.03
Attention UNet	82.3	70.7	94.0	4.88
UNet	78.7	66.2	95.3	5.22
FCN8s	74.1	60.5	99.4	5.86
VSAFU-Net	90.5	95.0	97.0	4.10

**Table 4:** SCS Net *vs.* State-of-the-art methods: Four-class classification.

**Reference**	**Method**	**ACC (%)**	**PRE (%)**	**REC (%)**	**F1-Score (%)**	**SPE (%)**
VSAG-HDL Net	SCS Net	95.5	92.4	94.9	93.6	98.3
Deep Neural Networks [38]	DNN	87.1	89.3	88.0	88.5	87.4
Ensemble ML Models [39]	ML	87.7	94.0	87.5	91.2	95.8
Corodet [40]	CNN	91.2	92.0	91.9	90.0	93.5
CovXNet [41]	CNN	90.20	90.8	89.9	90.4	89.1
SqueezeNet [42]	CNN	89.4	89.9	89.4	89.9	96.5
ShuffleNet [42]	CNN	91.6	91.4	91.6	91.4	97.3
COVIDetection-Net [42]	CNN	94.4	94.4	94.4	94.4	98.1
VIT [43]	Transformer	94.2	89.7	88.1	88.8	95.2
DenseNet [43]	CNN	97.5	96.2	94.8	95.4	97.9
DenseNet + VIT [43]	CNN + Transformer	97.5	96.25	94.9	95.5	97.9
DenseNet + VIT+GAP [43]	CNN + Transformer	97.8	96.9	95.6	96.2	98.1

## Data Availability

The data of current study are available from corresponding author, [B.E], on a reasonable request.

## LIST OF ABBREVIATIONS

ACC: Accuracy
AI: Artificial Intelligence
AUC: Area Under the Curve
BCE: Binary Cross-Entropy
CAD: Computer-Aided Diagnosis
CNN: Convolutional Neural Network
CLAHE: Contrast Limited Adaptive Histogram Equalization
COVID-19: Coronavirus Disease 2019
CT: Computed Tomography
CXR: Chest X-ray
DL: Deep Learning
DSC: Dice Similarity Coefficient
DNN: Deep Neural Network
DCNN: Deep Convolutional Neural Network
F1: F1 Score
FN: False Negative
FP: False Positive
GRAD-CAM: Gradient-weighted Class Activation Mapping
GAP: Global Average Pooling
GPU: Graphical Processing Unit
HD: Hausdorff Distance
IoU: Intersection over Union
KL: Kullback-Leibler Divergence
ML: Machine Learning
PACS: Picture Archiving and Communication System
PRE: Precision
REC: Recall
RT-PCR: Reverse Transcription Polymerase Chain Reaction
ReLU: Rectified Linear Unit
SCAN: Structure Correcting Adversarial Network
SCS: Sharpened Cosine Similarity
SGD: Stochastic Gradient Descent
SPE: Specificity
TN: True Negative
TP: True Positive
TSCN: Two-Stream Collaborative Network
VSA: Variational Spatial Attention
VSAFU-Net: Variational Spatial Attention Fusion U-Net
VSAG: Variational Spatial Attention-Guided
VSAG-HDL Net: Variational Spatial Attention-Guided Hybrid Deep Learning Network
ViT: Vision Transformer
